# Optimization of soil microbial fuel cell for sustainable bio-electricity production: combined effects of electrode material, electrode spacing, and substrate feeding frequency on power generation and microbial community diversity

**DOI:** 10.1186/s13068-022-02224-9

**Published:** 2022-11-16

**Authors:** Imologie Meshack Simeon, Alfons Weig, Ruth Freitag

**Affiliations:** 1 Process Biotechnology & Center for Energy Technology (ZET), University of Bayreuth, 95447 Bayreuth, Germany; 2 Genomics & Bioinformatics, University of Bayreuth, 95447 Bayreuth, Germany; 3 Department of Agricultural and Bioresources Engineering, Federal University of Technology Minna, PMB 65, Minna, Nigeria

**Keywords:** Sustainable bio-electricity, Soil microbial fuel cell, Optimization, Electrode spacing, Material, Substrate

## Abstract

**Background:**

Microbial fuel cells (MFCs) are among the leading research topics in the field of alternative energy sources due to their multifunctional potential. However, their low bio-energy production rate and unstable performance limit their application in the real world. Therefore, optimization is needed to deploy MFCs beyond laboratory-scale experiments. In this study, we investigated the combined influence of electrode material (EM), electrode spacing (ES), and substrate feeding interval (SFI) on microbial community diversity and the electrochemical behavior of a soil MFC (S-MFC) for sustainable bio-electricity generation.

**Results:**

Two EMs (carbon felt (CF) and stainless steel/epoxy/carbon black composite (SEC)) were tested in an S-MFC under three levels of ES (2, 4, and 8 cm) and SFI (4, 6, and 8 days). After 30 days of operation, all MFCs achieved open-circuit voltage in the range of 782 + 12.2 mV regardless of the treatment. However, the maximum power of the SEC–MFC was 3.6 times higher than that of the CF–MFC under the same experimental conditions. The best solution, based on the interactive influence of the two discrete variables, was obtained with SEC at an ES of 4.31 cm and an SFI of 7.4 days during an operating period of 66 days. Analysis of the experimental treatment effects of the variables revealed the order SFI < ES < EM, indicating that EM is the most influential factor affecting the performance of S-MFC. The performance of S-MFC at a given ES value was found to be dependent on the levels of SFI with the SEC electrode, but this interactive influence was found to be insignificant with the CF electrode. The microbial bioinformatic analysis of the samples from the S-MFCs revealed that both electrodes (SEC and CF) supported the robust metabolism of electroactive microbes with similar morphological and compositional characteristics, independent of ES and SFI. The complex microbial community showed significant compositional changes at the anode and cathode over time.

**Conclusion:**

This study has demonstrated that the performance of S-MFC depends mainly on the electrode materials and not on the diversity of the constituent microbial communities. The performance of S-MFCs can be improved using electrode materials with pseudocapacitive properties and a larger surface area, instead of using unmodified CF electrodes commonly used in S-MFC systems.

**Supplementary Information:**

The online version contains supplementary material available at 10.1186/s13068-022-02224-9.

## Background

Microbial fuel cells (MFCs) are bio-electrochemical devices capable of using electroactive bacteria (EAB) present in the environment to oxidize organic material and transfer electrons to an electrode as part of their metabolism to generate bio-electricity. It has been shown that practical usable energy can be derived from MFCs [1] with the help of environmental consortia [2, 3]. One such promising MFC is soil (S-MFC) with high potential for real-world applications [4, 5]. However, their low energy production rate and performance instability limit their application [6] in the real world. Therefore, optimization (both architectural and biological) is required to achieve optimal S-MFC system performance for direct practical applications. The individual aspects of S-MFCs, that are the focus of research are stack configuration, reactor design [7], electrode materials, inoculum, and operating conditions [8].

Unlike most electrical and chemical energy systems, which have a constant output power due to stable physical or chemical reactions, the output power of MFC is determined by the dynamic microbial activity [8] in the system. Therefore, the output power of a typical MFC is constantly changing depending on microbial activities and environmental conditions. The maximum power point (MPP) commonly reported is only a point-in-time estimate of the performance of MFCs. The usefulness of a bioenergy source requires long-term sustainability of its performance. The growth cycle of a bacterial culture generally has four distinct phases: the lag phase, in which bacteria are metabolically active but not dividing; the exponential phase, in which growth is exponential; the stationary phase, in which growth reaches a plateau as the number of dying cells equals the number of dividing cells; and the death phase, characterized by an exponential decline in the number of living cells [9, 10]. The electrical output of S-MFCs and most other MFCs follows the bacterial growth cycle. The stationary phase, during which maximum power is also achieved, lasts only a few seconds, minutes, and hours [11, 12] or at best a few days [13, 14], depending on the system. Therefore, an effective way to maximize the contribution of electrochemically active bacteria to the electrical performance of a bio-electrochemical system is to optimize the substrate, electrode material, and electrode potential [15, 16] as well as other influencing parameters [17].

The most important factors determining the availability of oxygen and substrate in a membrane-less single chamber S-MFC are the spacing of the electrodes and the frequency of treatment with a substrate. In addition, the anode–cathode distance plays an important role in ensuring efficient charge transfer between the electrodes to maintain the charge balance of the system [4]. The design of an optimization study of S-MFCs must, therefore, consider these factors and the treatment effects of their interaction. In addition, the type of electrodes influences the performance stability of fuel cells [18].

The effect of electrode spacing (ES) on the power output of a S-MFC at a constant substrate feed rate was recently studied [4]. While a smaller ES initially resulted in better performance, a reversal of the performance trend was observed with extended feeding. This observation was attributed to inappropriate intervals between substrate feedings (SFI) that negatively affected the performance of the S-MFC at lower ES, highlighting the need to find an appropriate SFI to continue to maximize performance at lower ES. Substrate depletion or limited carbon source [19] during batch operation results in a gradual decrease in S-MFC performance [20, 21]. Most MFCs designed for wastewater treatment operate at continuous flow to prevent substrate depletion [8]. However, continuous substrate feed is not possible in most S-MFCs due to their particular configuration [4]. Therefore, S-MFCs usually use a fed-batch mode, but the best time to feed must be found to prevent the substrate from becoming a limiting factor while reducing the effect of oxygen cross-over to the anodic region.

Electrode material (EM) is one of the most important material factors that influence the performance of a MFC. In addition to conductivity and large surface area, the biocompatibility of the electrode is very important for biofilm attachment [22] and efficient electron transfer. Carbon-felt (GF) electrodes are most commonly used in S-MFCs [4, 23, 24]. In addition to conductivity, corrosion resistance and ease of handling also contribute to the suitability of the CF electrode [25] for use in the soil–water environment. So far, both the cathode and anode of S-MFCs are mainly made of carbon-based materials, such as carbon brush, carbon cloth, carbon felt, graphite rods, and granulated carbon. However, the high resistance of the soil limits the performance of pure carbon electrodes in S-MFCs. Li et al. reported that an electrode made by rolling activated carbon and carbon black onto a stainless steel current collector [26] performed better than CF in a S-MFC. In a similar study, Simeon et al. showed that improving electrode capacity using stainless steel impregnated with carbon black resulted in three times better performance in an S-MFC compared to a CF electrode [27]. These important parameters affecting S-MFC performance have more often been studied independently without considering the interactions between two or more independent variables [28]. While many studies have focused on the material and architectural aspects of MFCs, the influence of the interaction of important boundary conditions on power output or the response of the diverse microbial community to a combination of these boundary conditions is generally still unclear in S-MFCs. Single-factor experiments usually lead to inconclusive and non-reproducible solutions, since MFCs are complex systems whose performance is determined by the interaction of multiple parameters [29]. Therefore, optimization of the S-MFC systems that incorporate multiple input parameters is necessary for improved performance [30, 31].

In this study, the interactive influence of three important parameters (SFI, EM, and ES) on the magnitude and stability of S-MFC power output was investigated to find the combination of these parameters that would achieve optimal performance of the S-MFC. The response of microbial community diversity was also investigated to determine the contribution of the microbial component of the S-MFC to the performance of different EMs. While the MFC consisted of complex microbial community diversity in all treatments, the results showed that among the factors studied, the electrode material was the most influential factor determining the performance and stability of the S-MFC for long-term bio-electricity harvesting for real-world applications.

## Results and discussion

### Individual treatment effects of electrode material, spacing, and feeding frequency

**Fig. 1:**
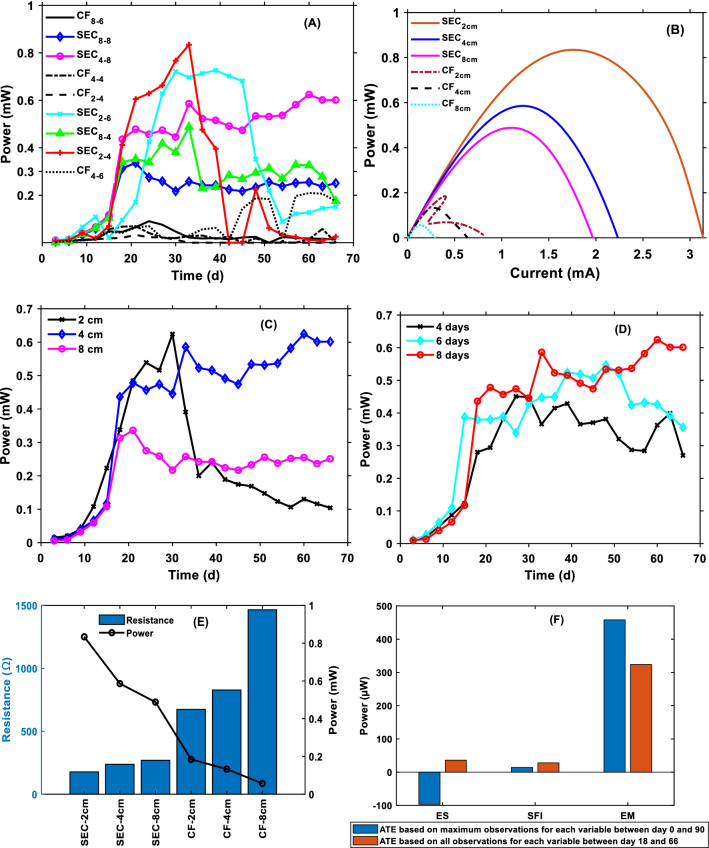
Individual treatment effects: (**A**) electrode type during the 66-day operating period—the legends (Fig. 1A, B) are described as X_y–z_, where *X* = electrode, *y* = ES and *z* = SFI, B best power curves based on a single point-estimation of the S-MFCs with different EM and ES; (**C**) effect of ES at 8 day SFI of SEC–MFC; (**D**) effect of SFI at 4 cm ES of SEC–MFC. **E** Power and internal resistance of S-MFC as a function of EM and ES

Figure 1 compares the performance of the S-MFC at different combinations of treatment levels. Only selected results around the optimal performance are shown to avoid unnecessary duplication (The power profiles of the S-MFC in terms of open circuit voltage (OCV) and power at all treatment levels are provided in the supplementary document). The MFCs built with SEC electrodes (SEC–MFCs) showed better performance than the CF–MFCs at all treatment levels (Fig. 1A, B, E). The better performance of the SEC–MFCs can be explained by their lower resistance (Fig. 1E). Based on a single-point estimation from the MPP, the overall best performance (834.5 µW or 251.5mW/m^2^) at a current of 1.77 mA and a resistance of 265 Ω for the SEC–MFC was obtained on day 33 at 2 cm ES due to its lower internal resistance, but this performance could not be sustained over a long operating period during substrate feeding probably due to oxygen transfer to the anode and possible short-circuit effects as the conductivity of the bulk electrolyte increased. For the CF–MFC, the best performance based on a single-point performance estimate was 234 µW (73.54 mW/m^2^) and was obtained at ES of 4 cm and SFI of 6 days, after 66 days. The average treatment effects (ATE) of each of the factors was calculated from Eq. 1, assuming complete randomization:

1 $$ATE=\frac{1}{n}\sum_{i=1}^{n}{(y}_{i}^{high level}-{y}_{i}^{low level})$$

where n is the number of observations and y is the response or measured observation.

Considering one factor at a time and neglecting the effects of other factors and their interactions, there were six observations (*n* = 6) for each level of ES and SFI (Table 1). The ATE of ES between 2 (low level) and 8 (high level) was − 96.4 µW using only the maximum responses at these levels. This means that the maximum power decreased by an average of 96.4 µW when the ES was increased from 2 to 8 cm. This indicates that a lower ES produces higher power in S-MFC when other influencing factors are constant. The better performance at lower ES is due to lower R_int_ as can be established from Fig. 1E. From the result shown in Fig. 1E, it can be deduced that for the SEC electrode, the R_int_ increased by 25.4% and the power decreased by 42% when ES was increased from 2 to 4 cm. Similarly, when ES was increased from 4 to 8 cm, R_int_ increased by 11.8% and power decreased by 20%. In contrast, for the CF electrode, increasing ES from 2 to 4 cm and from 4 to 8 cm resulted in a percentage increase in R_int_ of 18.6 and 43.5, respectively, and a percentage decrease in the power of 39.1 and 133.3, respectively. This shows that the performance of the S-MFC in this study depended on the different variables and the resulting interactive effects. When the SFI was increased from 4 to 8 days, the ATE was 14.2 µW, indicating that feeding too frequently had a negative effect on S-MFC performance. To gain further insight into the treatment effects of the variables, including their interactions, all observations between days 18 and 66 [when substrate feeding occurred and most MFCs reached stability (Additional file 1: Fig. S1)] were used to calculate the ATE (Fig. 1F). It can be concluded that performance was best at an ES of 2 cm without the influence of SFI, but that with substrate feeding, higher ES resulted in a better and more stable performance of the SMFC. With an ES of 2 cm, performance was more stable at the SFI of 6 days, but performance stability decreased when SFI was increased to 8 days. The 2-cm ES also showed the fastest response to substrate feeding, while the 8-cm ES showed the slowest. The SFI of 8 days resulted in a more stable performance with 4 cm and 8 cm ES (see Additional file 1: Fig. S1 for details). For the EMs, the ATE showed that the power of the S-MFCs increased by 458.16 µW by switching from CF to SEC electrodes during the operating period when the average of the maximum observation at each level was used to calculate the ATE. However, when the average of all observations within the stability window (days 18 and 66) was used, the ATE was 324.05 µW (Fig. 1F), proving the superiority of the SEC electrode over the CF electrode in the S-MFC in terms of both magnitude and stability.

**Table 1:** Experimental treatments (ES, SFI, and EM) and response in maximal power for each MFC. The responses are presented as mean ± standard deviation for each MFC

MFC	Factor 1 A: ES (cm)	Factor 2 B: SFI (day)	Factor 3 C: EM	Response P (µW)
1	8	6	SEC	471.6 ± 68.1
2	2	6	CF	10.33 ± 21.0
3	2	8	SEC	248 ± 164.7^a^
4	4	6	SEC	438.9 ± 62.3
5	8	8	CF	102.7 ± 42.6
6	8	4	CF	20.1 ± 18.1
7	4	8	CF	69.3 ± 66.4
8	4	4	SEC	359.21 ± 58.5
9	2	4	CF	8.3 ± 12.2
10	8	6	CF	33 ± 22.8
11	8	8	SEC	253.2 ± 30.4
12	4	8	SEC	516.7 ± 54.4
13	4	4	CF	24.3 ± 22.3
14	2	8	CF	7.7 ± 12.9
15	2	6	SEC	397 ± 264.2^a^
16	8	4	SEC	313.1 ± 71.9
17	2	4	SEC	304.3 ± 299.2^a^
18	4	6	CF	109.8 ± 75.2

^a^The large standard deviations at 2 cm ES showed that the MFCs’ performance was most unstable at this electrode spacing

Using the ATEs, it can be concluded that the treatment effects of the individual factors are in the order of SFI < ES < EM. Irrespective of the treatment levels of ES and SFI, the SEC–MFC exhibited better performance than the CF–MFC. The higher resistance and occurrence of power overshoot with the CF–MFC (Fig. 1B) testifies to the poor performance of the unmodified CF electrode in S-MFC. The performance trend shown in Fig. 1E has shown that the total internal resistance of SMFCs mainly depends on the type of electrodes used as anode and cathode, as well as the electrode spacing. However, by considering only the ATEs of the individual factors, it is difficult to find a single solution to the optimization problem, since the interactive effects have not been fully considered. The sustainability of the power at each level of ES and SFI was also not accounted for by considering only the maximum performance. Therefore, to obtain a single solution which is the point of convergence of all the levels of the factors including their interaction, the average performance between days 18 and 66 when most of the MFCs attained stable performance was used to model the power as a function of the ES and SFI for each electrode material.

### Optimization responses and interactive effects

Table 1 shows the average responses obtained under different combinations of the selected variables in the experimental design. The response (power) used in the model for each run was obtained from experimental data averaged over 17 actual measurements of the MFCs between days 18 and 66.

**Fig. 2.**
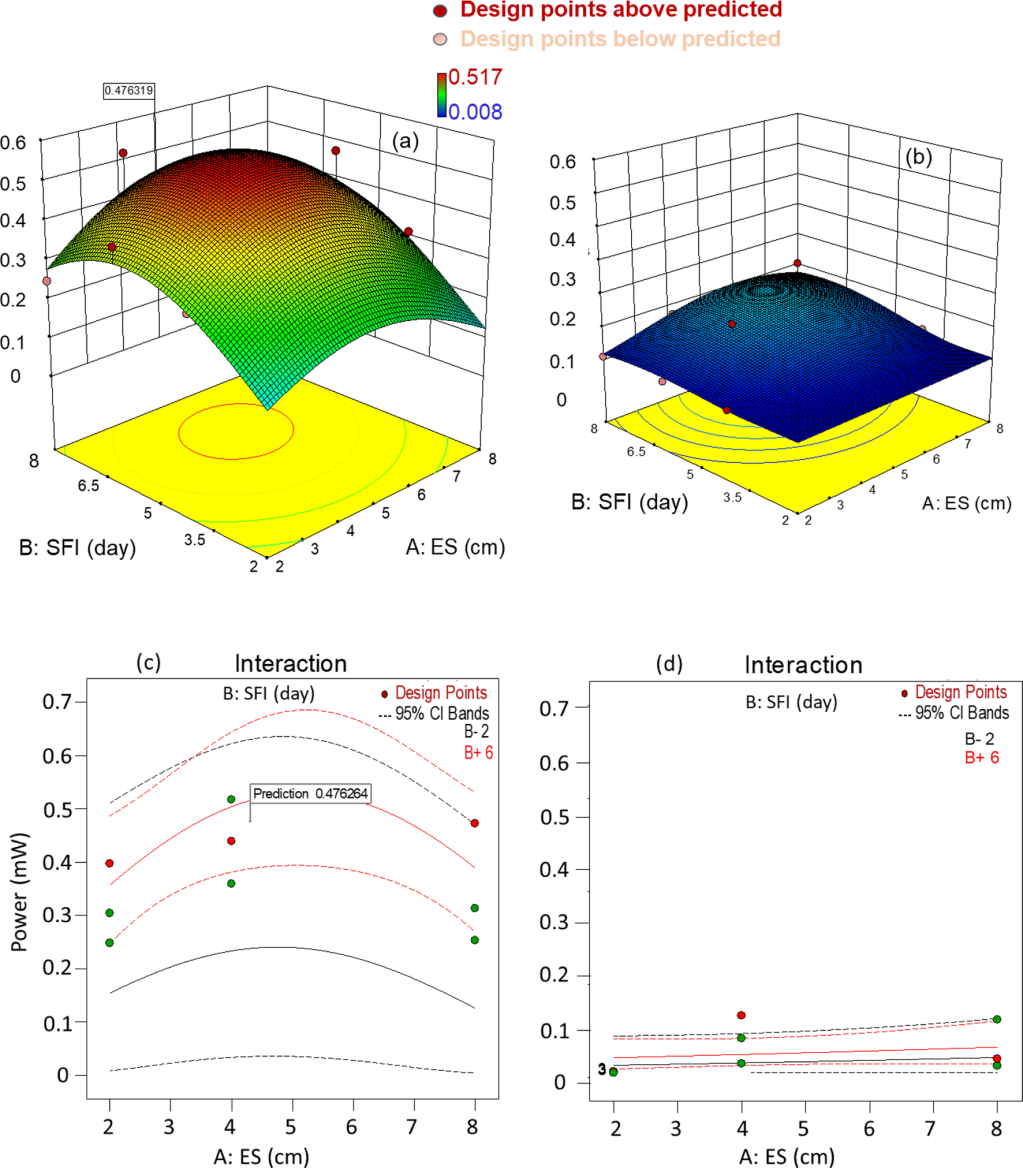
3D plots of the absolute power response to ES and SFI with (**a**) SEC and (**b**) CF electrodes. Interactive effect of ES and SFI with (**c**) SEC, (**d**) CF electrodes. The green markers represent actual responses below and above the optimal prediction point

Figure 2 shows the response surface three-dimensional (3D) plots generated by optimizing the parameters. The influencing variables used are ES (A), SFI (B), and EM (C). The responses are shown in two different graphs (Fig. 2a, b), since factor C is categorical and there can be no possible interaction between the two different EMs. The quadratic model (Additional file 1: Table S1) created from the optimization system using Design expert was significant with an R^2^ value of 0.9568 and an adjusted R^2^ value of 0.9135, which is a reasonable match to the predicted R^2^ value with a difference of less than 0.2.

The F value of the model also shows that the probability of error due to noise is only 0.01%, indicating that the model can be used to navigate the design space. EM and the squared value of ES were the only model terms that were found to be significant (significance level: α = 0.05). Details of the models and the analysis of variance for the model terms are provided in the supplemental document (Additional file 1: Table S1). Response surface plots were created to examine the effects of each parameter and to determine if there was a significant interaction between any two of the parameters. The existence of a second-degree polynomial relationship between power and SFI and power and ES is evident for the SEC electrode (Fig. 2a). The response surface for CF, on the other hand, shows a nearly parallel relationship, revealing no significant interaction between ES and SFI. The plots of the interactive effects of ES and SFI shown in Fig. 2c produced non-parallel lines, indicating that the effect of ES on maximum power depends on the level of SFI. There was a point of convergence for the values of the two discrete variables that gave the range of the best solution for the combination of the two discrete variables for SEC and CF (Additional file 1: Table S2). The overall best solution for 2 combinations of ES and SFI yielded 0.476 mW (P) at 4.31 cm (ES) and 7.4 days (SFI) for the SEC electrode; and 0.113 mW (P) at 6.4 cm (ES) and 7.2 days (SFI) for CF. Better performance was obtained with larger ES when SFI was between 5.5 and 6.5 days, while the optimal performance (0.476 mW) predicted by Design Expert was obtained with SEC at an ES of 4.3 cm and SFI of 7.4 days.

### Impedance spectroscopy, specific surface area, and conductivity of electrodes

**Fig. 3:**
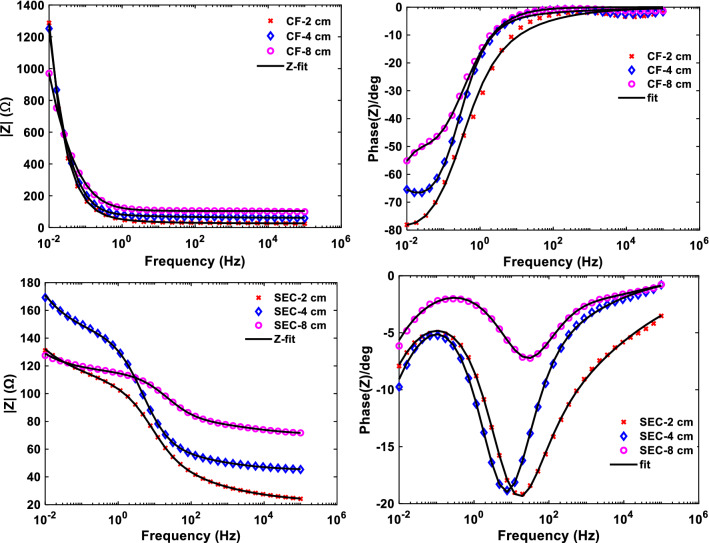
Bode impedance characteristics of the S-MFCs with different EM and ES: impedance as a function of frequency (left) and Phase shift as a function of frequency (right)

The impedance characteristics of the electrodes have a great influence on the electrodes. Although the LSV results (Fig. 1) showed that the S-MFCs operated with CF had a higher R_int_, the distribution of this internal resistance could not be determined. Therefore, EIS was performed to determine how the two different electrodes affected the impedance and capacitive characteristics of the S-MFCs and which component of the impedance parameters contributes most to the R_int_. The EIS was performed during the steady state of the best MFCs based on the two EMs at SFI of 6 days and ES of 2, 4, and 8 cm. The Bode plots of the EIS are shown in Fig. 3. Table 2 shows the calculated EIS parameters by simulating the measured data from the Nyquist plot (Additional file 1: Figs. S2 and S3) with an electrical equivalent circuit (Fig. 9a).

**Table 2:** EIS parameters fitted with an equivalent circuit

EM–ES (cm)	R_Ohmic_ (Ω)	C (F)	R_ct_ (Ω)	σ (Ω.s^−1/2^)
CF-2	28	4.31E–21	1375.2	36.7
CF-4	60.7	1.70E–12	1352	16.1
CF-8	103.3	2.94E–14	986.6	19.5
SEC-2	21.2	1.14E–04	97.7	4
SEC-4	44.4	2.90E–05	98.7	6.6
SEC-8	70.2	3.86E–06	45.5	3.2

**Fig. 4:**
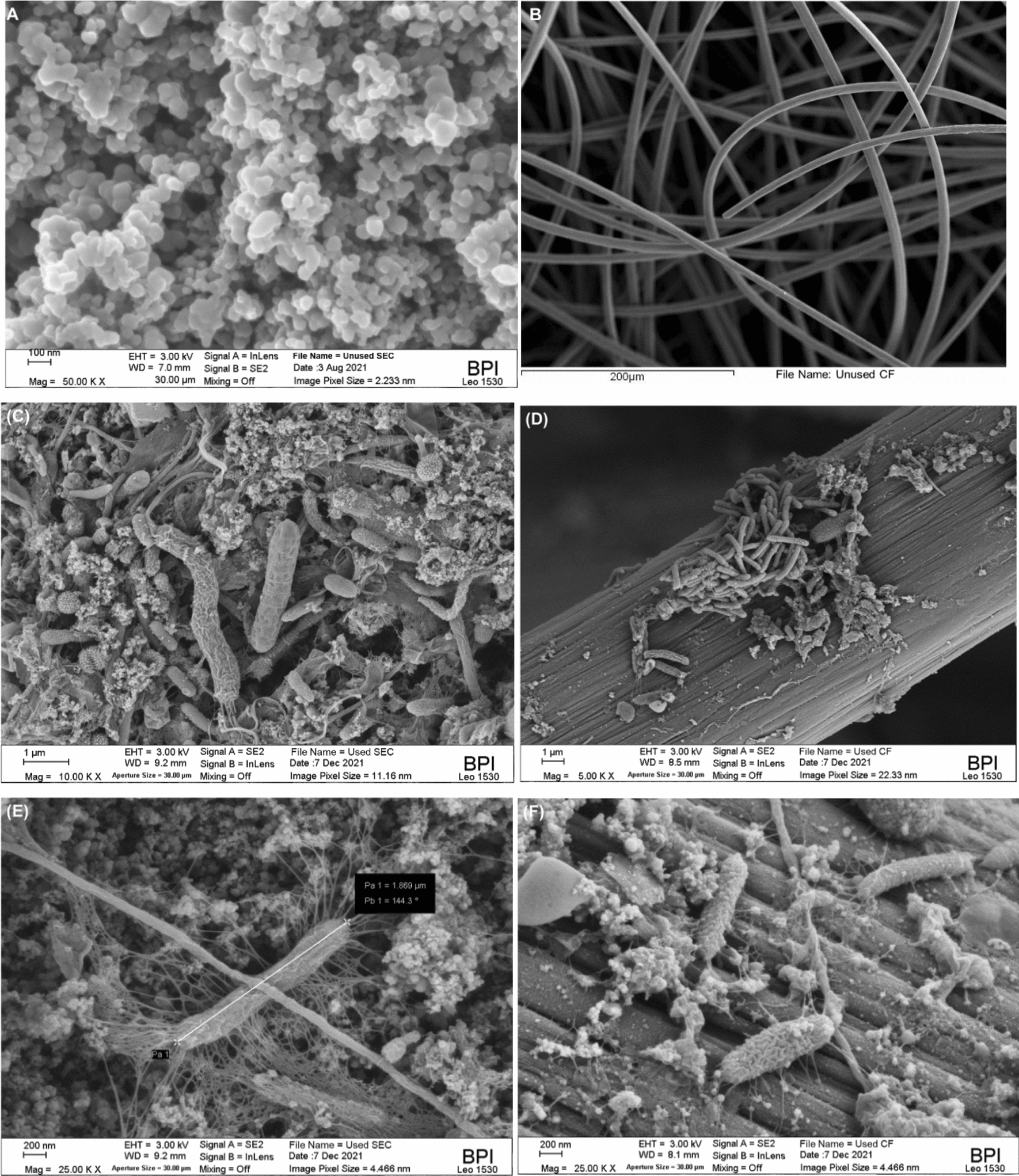
Biofilm interaction with the electrodes: unused (**a**) SEC, (**b**) CF; Used with biofilm (C&E) SEC, (D&F) CF

The SEC-MFCs and CF-MFCs electrodes produced comparable Ohmic resistance (R_Ohmic_) values, as shown in Table 2. In general, R_Ohmic_ comprises the resistance caused by the conductivity of the bulk electrolyte (in this case mud), the biofilm, the catholyte, and the electronic resistance of the electrodes [32] due to current collectors. One factor that could influence the low performance of the CF electrode is the current collector used, in this case, titanium wire. The SEC electrode was connected by extending the base stainless steel mesh, which may have created multiple contact points and thus a more efficient electron transfer path. However, to avoid poor electrical contact between the current collector and the fibers of the CF, the wire was wrapped around the electrode. The CF-MFCs produced comparable R_Ohmic_ to the SEC–MFC at all ES levels, suggesting that their poor performance was not related to the poor contact between the current collector and the electrode. The proportionality of R_Ohmic_ to ES can be seen for both electrodes. This illustrates why better performance was obtained at lower ES when the influence of SFI was minimal. The CF-MFCs were dominated by charge transfer resistance and slow diffusion processes at low frequencies as indicated by the high diffusion coefficient (σ) (although the Warburg impedance component was used in the equivalent circuit, only the coefficient can be estimated with the EC-Lab software). The SEC–MFCs have comparable R_Ohmic_ values but are characterized by lower charge transfer resistance and higher capacitance. The pseudocapacitive behavior of the SEC–MFCs is well-illustrated by the peaks in the phase shift of the Bode plot spectrum (Fig. 3). With the SEC electrode, the capacitance value was higher at lower ES, suggesting that the S-MFC built with SEC electrode could function as a self-charging supercapacitor with the electrodes as parallel plates and the mud as the dielectric. The pseudo-capacitance of the SEC electrodes contributed to charge storage and resulted in better performance of the SEC–MFC compared to the CF–MFC which lacks the capacitive properties [27]. The larger charge transfer resistance of CF may have resulted from the large void spaces between the conductive fibers of the CF electrodes as can be seen in the SEM images (Fig. 4) and the low capacitive property (Table 2). The resistance characteristics of the S-MFCs obtained From LSV and EIS are very similar showing clearly that the performance of the unmodified CF electrode in S-MFC is limited by high R_int_. In addition, the EIS result showed that higher resistance to charge transfer and mass transport were the major resistive components of the CF–MFC that contributed to its poorer performance compared to the SEC–MFC.

Interestingly, the investigation of the conductivity of the electrodes revealed that the CF electrode has a higher conductivity due to its lower resistivity than the SEC electrode (Table 3). The lower conductivity of the SEC is attributed to the non-conductive polymer binder used to bind the conductive CB to the stainless steel mesh. Higher conductivity alone did not lead to better performance of the CF electrode. Specific surface area is another important factor that determines the performance of electrodes in a bioelectrochemical system. The specific surface area of the SEC electrode was more than four times that of CF (Table 3). Consequently, the SEC electrode provided a larger reaction surface for microbial metabolism, resulting in better performance. This result is in agreement with the report of Bataillou et al. who noted that the surface area of electrodes and other surface properties play a vital role in bacteria adhesion to the electrode surface and consequently affect the electron transfer kinetic between microbes and electrodes [33].

**Table 3:** Physicochemical properties of the electrodes

parameters	SEC	CF
Resistivity (Ω.cm)	0.21 ± 0.01	0.13 ± 0.001
Conductivity (Ωcm^−1^)	4.87 ± 0.23	7.96 ± 0.04
Specific surface area (m^2^/g)	13.3 ± 0.05	3.05 ± 0.19
Weight of sample (g)	0.68 ± 0.01	0.13 ± 0.003

^a^measurement was made in triplicate and values were reported as mean + standard deviation

### Electrode–microbes morphological characteristics

Since the electrode type showed the greatest effect in this study, SEM was conducted on the electrode before and after use to understand the morphological characteristics of the electrode and the microbes–electrode interactions. Figure 4 shows the structure of the unused electrode and the used electrodes showing the physical interaction of the biofilms with the electrode’s surfaces.

The SEM analysis revealed that the SEC electrode consisted of mainly mesoporous materials and the CB particles were systematically arranged and bonded. The average diameter of the carbon particles on the electrode surface ranges from 20 and 90 nm. The CF electrode, on the other hand, consists of much larger carbon-felt fibers loosely interconnected with much larger void spaces (> 50 µm) between the felts. Figure 4 shows that bacteria with similar morphological characteristics are detectable on both EMs. This implies that both EMs are biocompatible and support the formation of a robust biofilm, which is necessary for the generation of bioelectricity. This assertion is also supported by the fact that both SEC–MFCs and CF–MFCs achieved similar values of open-circuit potential (Additional file 1: Fig. S1A, B). Therefore, the power losses are mainly due to electron transfer within the electrodes, as can be explained by Eq. 2. The better performance of the SEC electrode compared to the CF electrode in all treatments can be attributed to accelerated electron transfer due to the lower resistance and larger surface area of the SEC electrode. The large gap between the fibers of the CF electrode may have contributed to inefficient electrode transfer, as different microbial communities mainly use direct electron transfer between the cells and the electrodes. Physical examination of the electrodes after use revealed that the large voids in the CF electrode were mainly occupied by sand particles, which could have caused the large resistance to charge transfer. As seen from the SEM images, the strong physical interaction (attachment) between the electroactive biofilm and the nanoparticles of CB could result from the use of CB and epoxy paste as a carbon source by the microorganisms. Moreover, the close association of the carbon atoms of the conductive CB provides an efficient electrode transfer pathway between the microbes and the electrodes. The result of the present study is consistent with the findings of Yang et al. who reported that the performance of MFCs can be improved twofold due to accelerated electron consumption when the electrode is modified with interposed mesoporous carbon particles [34].

### Microbial community compositional abundance and diversity

**Fig. 5:**
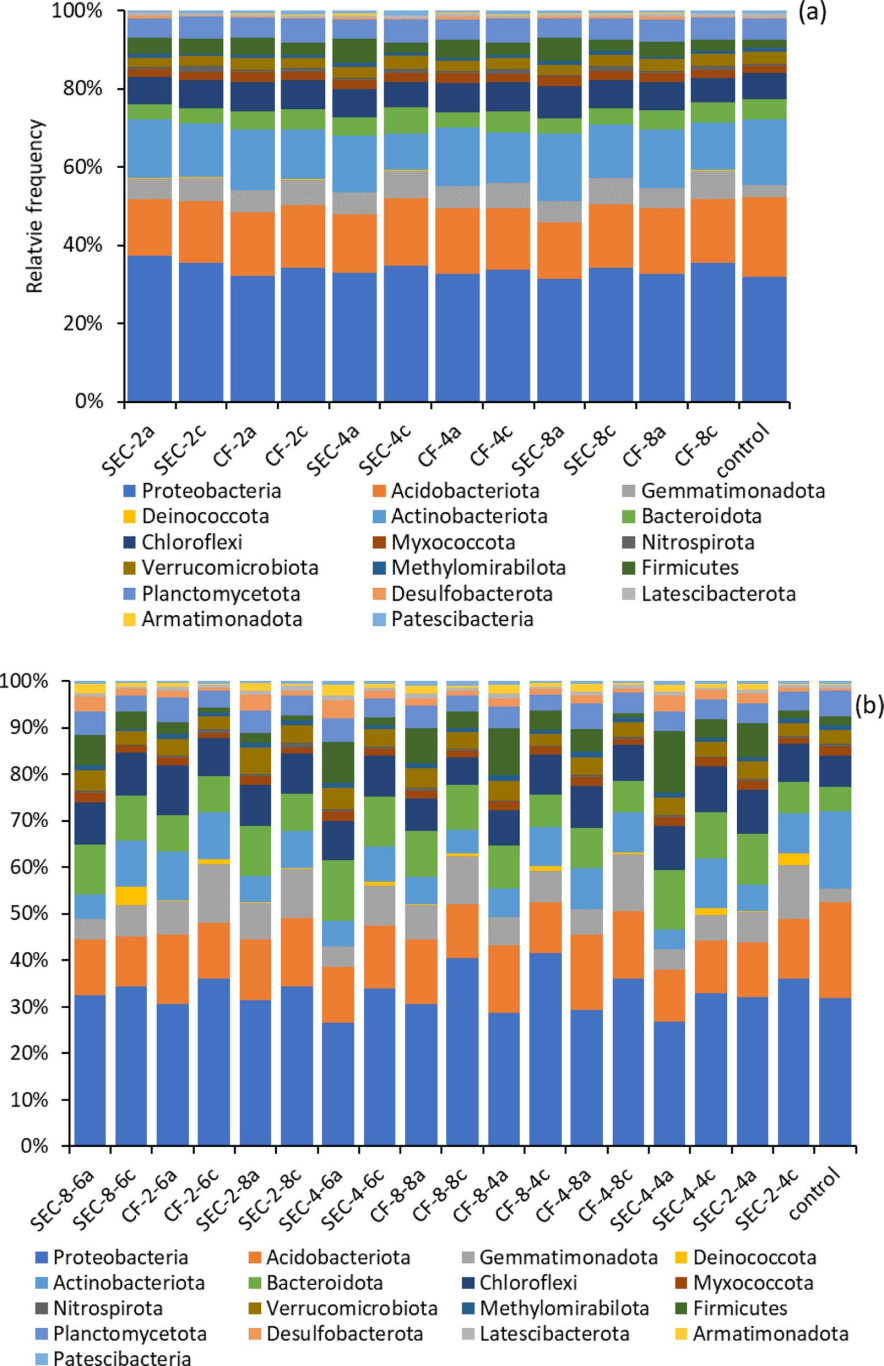
Taxonomic distribution of the most abundant 16S rDNA microbial community profile at the Phylum level: (**a**) MPP (40 days), (**b**) 90 days. The ‘a’ and ‘c’ in the *x*-axis labels indicate anode and cathode, respectively

The microbial community input in this study was considered an uncontrolled variable, since the natural mixed microbial community of the soil was used. For a better understanding of how the treatments influenced the microbial community and the power generation, 16S rDNA gene amplicon sequencing was performed on purified DNA samples from the anode, cathode, and MPP. Figure 5 presents the relative abundance of the most abundant phyla that were found to be at least 1% of the total bacterial taxa counts. The taxa bar plot for all the identified phyla is given in the supplementary document (Additional file 1: Fig. S4).

No significant difference was found in the abundance of the phyla across treatments (SFI and EM) but a significant difference was found between anode and cathode. Apart from *Acidobacteriota* and *Chloroflexi,* all other Phyla show a significant difference in relative abundance between anode and cathode and between the time of sampling but not between CF and SEC at the same sampling time as shown in the PERMANOVA analysis given in Additional file 1: Table S3.

Some phyla that showed no statistically significant difference in relative abundance between anode and cathode during the MPP showed significant differences in abundance at the end of the study. *Planctomycetota*, for example, which were relatively more abundant at the cathode during the MPP, were more abundant at the anode at the end of the study. This is not surprising, however, as members of this phylum are known to adapt to both aerobic and anaerobic conditions [35].

*Proteobacteria* were the most abundant phylum in all treatments—30.7–36.69% (MPP) and 25.31–40% (90 days). Their relative dominance at the anode, cathode, and the point of maximum output suggests that members of this phylum were functionally stable, able to adapt to the treatments, and likely contributed most to the overall electroactivity. *Proteobacteria* generally represent the largest strain of EAB that dominate the microbial communities of MFCs and are capable of directly transferring electrons to the electrodes [36, 37]. The *Proteobacteria* found in this study belonged predominantly to the class of *Alphaproteobacteria* and *Gammaproteobacteria*, whose electroactivity in MFC systems is well-documented [36]. At the family level, *Nitrosomonadacea*, *Xanthobacteriaceae*, *Xanthomonodaceae*, and *Sphingomonadaceae* were the 20% most abundant members of the *Proteobacteria* and were the same at the anode, cathode, and MPP. The family *Rhodocyclaceae*, which also belongs to the phylum *Proteobacteria*, was not identified in the original soil used to inoculate the MFCs. It was among the lowest 5% of the group with the lowest abundance at MPP but became one of the 10% most abundant families after 90 days. Members of this family have the special ability to “degrade a wide range of carbon sources, including many aromatic compounds, using oxygen, nitrate, chlorate, perchlorate, selenate, and other electron acceptors, as well as sulfur-oxidizing chemoautotrophs, methylotrophs, and anaerobes that perform propionic acid fermentation” [38]. Some of the members of this family that degrade aromatic compounds have the potential to biodegrade organic wastes and thereby remediate polluted environments [38]. The appearance of strains not originally found in the soil may be related to the particular ability of MFCs operated with different heterogeneous microbial consortia to self-optimize their overall performance in terms of power generation and pollutant degradation.

In addition to *Proteobacteria*, phyla *Acidobacteriota* and *Chloroflexi* also showed similar characteristics in terms of stability of relative abundance in the control, anode, and cathode. One phylum that exhibited a unique characteristic in this study was the *Firmicutes*. The relative abundance of this strain in the MFCs was significantly increased compared to the original soil. It was more abundant at the anode compared to the cathode, and its abundance also correlated positively with the performance of the MFCs at the time of sampling. Members of this phylum found at the anode are closely related to the *Christensenellaceae*, *Gracilibacteraceae*, and *Anaerovoracaceae* families, all of which belong to the *Clostridia* class. *Firmicutes* have a thick cell wall that allows them to adapt to harsh environmental conditions. *Clostridium butyricum* and *Methylomusa anaerophila* are strains belonging to *Firmicutes* that have been successfully isolated and used in MFCs with high potential for bioelectricity generation [36].

**Fig. 6:**
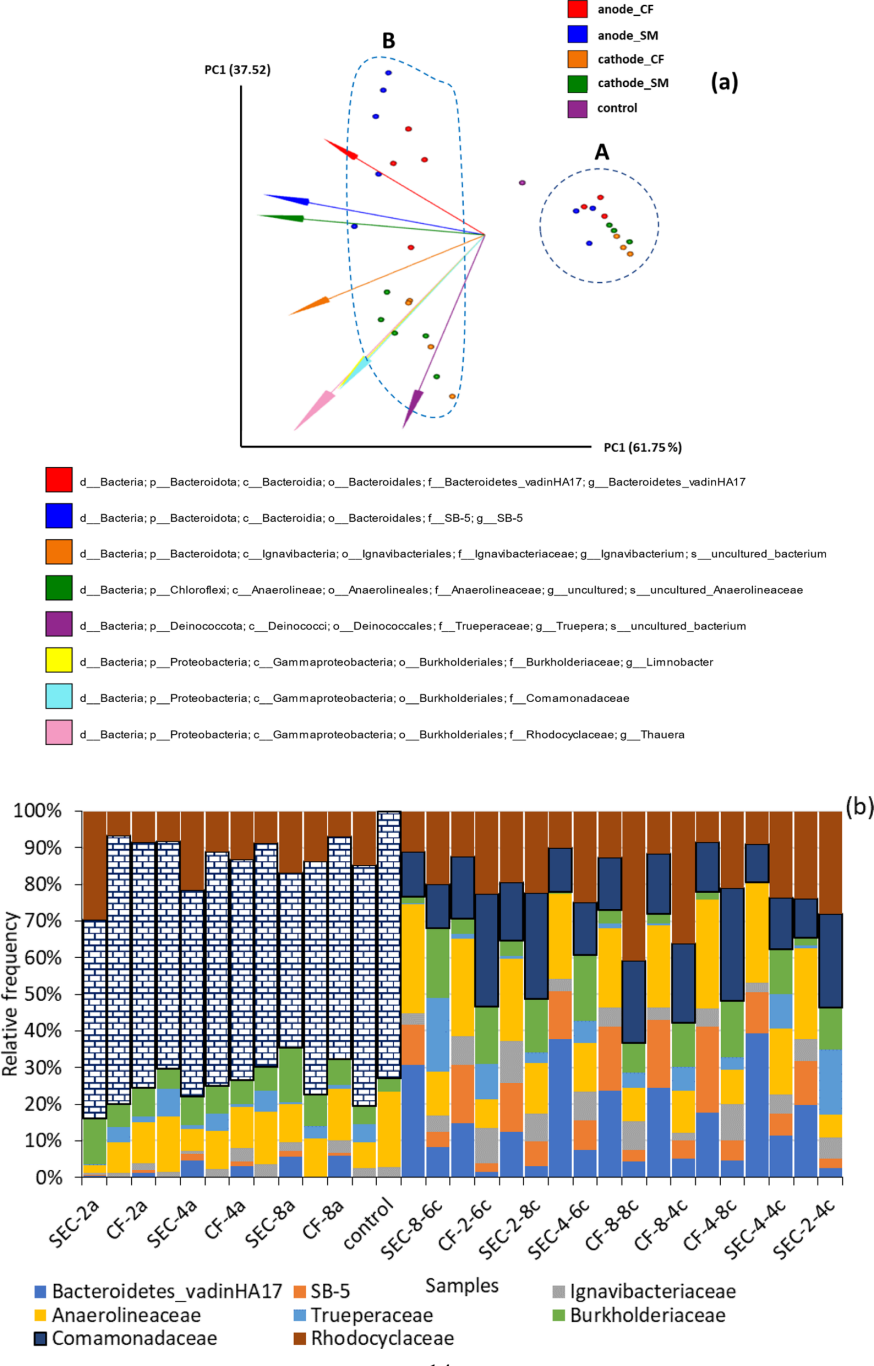
**a** RPCA comparing samples taken on day 0 (control), day 40 (A), and day 90 (B) from the anode and cathode of the S-MFCs at the family level. The arrow vectors show the microbes that most significantly contributed to the observed variations. **b** Relative abundance of the most diverse microbial groups in the treatments. The patterned bars represent the *Comamonadaceae* family for the MPP group

At the family level *Desulfocapsaceae*, *Desulfuromonadaceae*, and *Geobacteraceae* belonging to the phylum *Desulfobacterota* were among the 20% most abundant population with *Geobacteraceae* being most abundant at the anode. The family *Geobacteraceae* was initially located in the order *Desulfuromonadales* in the *Deltaproteobacteria* subclass of the phylum *Proteobacteria* [39] but is now named in the recently enacted phylum *Desulfobacterota* which encompasses sulfate-reducing and related fermentative and syntrophic lineages [40]. Some members of the family *Geobacteraceae* can directly transfer electrons to electrodes using electrochemically active redox enzymes, such as cytochromes, on their outer membrane [41]. *Geobacter* species that belong to this family are among the most widely studied group of bacteria within microbial fuel cell studies due to their ability to make electrical contacts with extracellular electron acceptors and other organisms [42, 43]. Although the difference in the performance of the electrodes in this study cannot be directly associated with the difference in the microbial populations of the different EMs, the 16S rDNA gene amplicon sequencing revealed complex microbial diversity, including members of the well-known EAB, that showed significant compositional changes at the anode, cathode, and MPP, as illustrated with the Robust Aitchison principal component analysis (RPCA) plot (Fig. 6a).

The observed variations in composition and relative abundance between 40 days (MPP) and 90 days occurred mainly along the first principal component, which explained 61.75% of the variations, while the variations between the anode and the cathode occurred along the second principal component. Figure 6a shows that eight families belonging to four different phyla contributed most to the observed variations. Figure 6b compares the relative abundance and composition of these bacteria between the anode and cathode and the time of sampling.

*Comamonadaceae* was the most abundant family in this group in the control sample and the MPP accounting for between 47.73% (SEC-8a) to 72.89% (control) in abundance relative to other families. This family was the most contributor to the observed variation in relative abundance between MPP and day 90. After 90 days, the relative frequency of this family decreased to 10.25–30.6%. It should be noted that the percent abundance stated here is only relative to the group here and not to the whole Taxa counts. Most of the denitrifiers reported in solid-phase denitrification specifically belong to the family *Comamonadaceae* in the phylum Proteobacteria [44]. Members of this family may have been actively involved in nitrate removal from the S-MFC because of their reported activeness in the solid-phase denitrification process. *Comamonadaceae* belonging to the order *Burkholderiales* is a large family of bacteria that include aerobic organotrophs, anaerobic denitrifiers and Fe^3+^-reducing bacteria, hydrogen oxidizers, photoautotrophic and photoheterotrophic bacteria, and fermentative bacteria. Most are environmental bacteria from water and soil habitats [45].

Many MFCs studies have demonstrated that members of the *Comamonadaceae* family are electroactive and dominated the microbial communities found in the MFCs. *Comamonadaceae* family can generate electricity in MFCs in the absence of oxygen and nitrate [46, 47]. Timmers et al. (2012) reported that members of this family dominated a high-current plant microbial fuel cell [47]. Being facultative anaerobic denitrifiers, their dominance at the anode and cathode at the MPP show that they were active at both electrodes. After 90 days, the relative abundance of this family decreased to between 10.25% and 30.6% across the groups. The current generation from the MFCs also dropped at this point suggesting that *Comamonadaceae* may have played an active role in the electricity generation.

At 90 days, more anaerobic families were found at a higher relative frequency at the anode, while more aerobic families were more relatively abundant at the cathode. For instance, *Anaerolineaceae*, which comprise obligate anaerobes were more abundant at the anode, while members of *Trueperaceae* and *Burkholderiaceae* that are either obliquely aerobic or facultatively anaerobic were relatively more abundant at the cathode. This difference in abundance and composition of microbial communities at the anode and cathode was necessary to maintain a potential difference for electron flow, as the microbial communities of these electrodes must be able to perform complementary reactions—electron donation and uptake [3]. The families *Bacteroidetes_vadinHA17* and *SB-5* constituted the compositional diversity observed in the microbial communities, because they were not present in the control sample and at the cathode in the MPP sample but were found at both the cathode and anode after 90 days. This compositional diversity consisting of both aerobic and anaerobic microbes showed that the complex microbial communities in S-MFCS synergistically benefitted the overall electroactivity. Although this analysis revealed complex microbial diversity that showed significant compositional changes at the anode, cathode, and MPP, no significant change in the microbial community could be associated with the two EMs.

### Reproducibility and applicability of the power of soil microbes

**Fig. 7:**
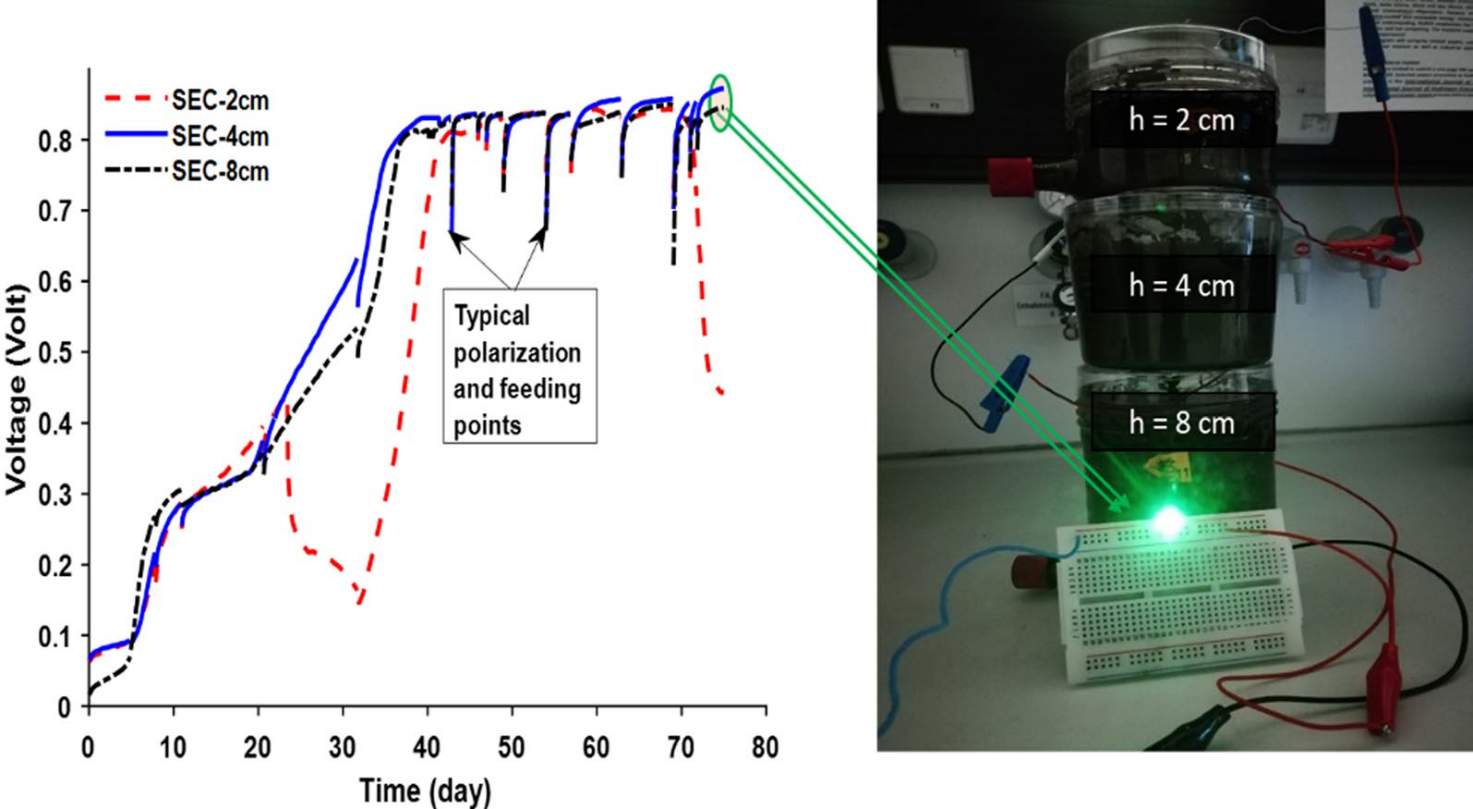
Reproducibility and practical implementation of the power of soil microbes

Stability and reproducibility are serious issues related to MFC performance that are rarely considered in most studies. However, biofuel cells must produce a stable level of sufficient power to be useful for driving electronic loads. To check the reproducibility of the obtained results, a new experiment was set up and performed by eliminating the factors that did not significantly affect the performance of the MFCs based on the optimization study. Here, only the SEC electrode was used at the 2 cm, 4 cm, and 8 cm ES, but no fixed SFI was applied. Feeding was only performed when a drop in performance was observed rather than a regular feeding operation. Figure 7 shows the reproducibility and applicability of the optimized S-MFCs.

The MFCs in this repeated experiment were operated in the open circuit but occasionally polarized with LSV at 1 mv/s and 0.1 mv/s to provide a means of electron transfer and charge balance between the anode and cathode. Feeding was performed only when a decrease in performance was observed. After about 30 days of operation, evidence of matured biofilm could be established as the MFCs irrespective of ES produced stable OCV in the range of 840.2 ± 8.4. It can be seen (Fig. 7) that comparable voltages can be obtained with different ES. However, the effect of oxygen transfer on the anode was more noticeable at 2 cm ES, which makes it difficult to maintain the performance of S-MFC at this ES when feeding is performed with wastewater. Stacking small units of MFCs is one of the most efficient strategies for obtaining useful bioelectricity [7]. The synergistic performance of the SEC–MFCs in series connection at different ES to drive light-emitting diodes (Fig. 7) makes the S-MFC an effective biofuel candidate for developing environmental biosensors powered by indigenous environmental microbes [2, 4].

## Conclusions

The study aimed to optimize a soil microbial fuel cell for sustainable bioelectricity production. The optimization result shows that between two tested electrode materials (EMs)–carbon felt (CF) and modified stainless steel (SEC), the SEC produced the best sustainable and practical usable performance at an electrode spacing of 4 cm and a feeding frequency of 8 days. The reproducibility test showed that feeding is best when performance deterioration is observed, rather than at a fixed feeding frequency. Sequencing of 16S rDNA gene amplicons from DNA samples from the anode, cathode, and point of maximum power revealed complex microbial diversity consisting of members of the known electroactive microbial communities and many others. *Proteobacteria* was the most abundant Phylum in all treatments, but the Firmicutes Phylum showed microbial abundance with a positive correlation to the power of the MFCs. There was no statistically significant difference in composition and abundance of the microbial communities between the two EMs but significance was found between anode and cathode and the time of sampling. It can, therefore, be reaffirmed that the soil contains a good mixture of EAB capable of direct electron transfer and can be harnessed for the production of useful bioelectricity. The difference in the performance at the studied level of treatments was not due to the difference in microbial community diversity but mainly due to the impedance and capacitive properties of the EMs. The better performance of the SEC electrode is attributed to more efficient electron transfer between the EAB and the electrodes due to the larger specific surface area providing a better reaction site for the microbe–electrode interaction. Although electrode spacing and substrate feeding influenced the performance of the S-MFCs in this study, electrode material was the most influential factor that needs to be further improved for sustainable and practical bioelectricity generation from the soil microbial fuel cell.

## Materials and methods

### Experimental design for multiple factor optimization

An optimal custom experimental design (Design Expert 10.10) was used to include all variables of interest in a single design and to examine the effects and interactions of treatment between variables. The input variables of interest in this study included two numerical factors and one categorical factor. The numerical factors were substrate feeding interval (SFI) and electrode spacing (ES), both at three treatment levels. The ES levels were 2, 4, and 8 cm, while the SFI levels were 4, 6, and 8 days. The whole experimental design comprised a total of 18 runs, which were randomly divided into two blocks of 9 runs each, forming 9 replicates for each electrode material and 3 replicates for the ES before feeding was started. The categorical factor is electrode material (EM) with two treatment levels: Carbon Felt (CF) and Stainless Steel/Epoxy/Carbon Black Composite (SEC). The measured response for all levels of treatment was power (P).

### Construction of the electrodes; assembly and operation of the S-MFCs

**Fig. 8:**
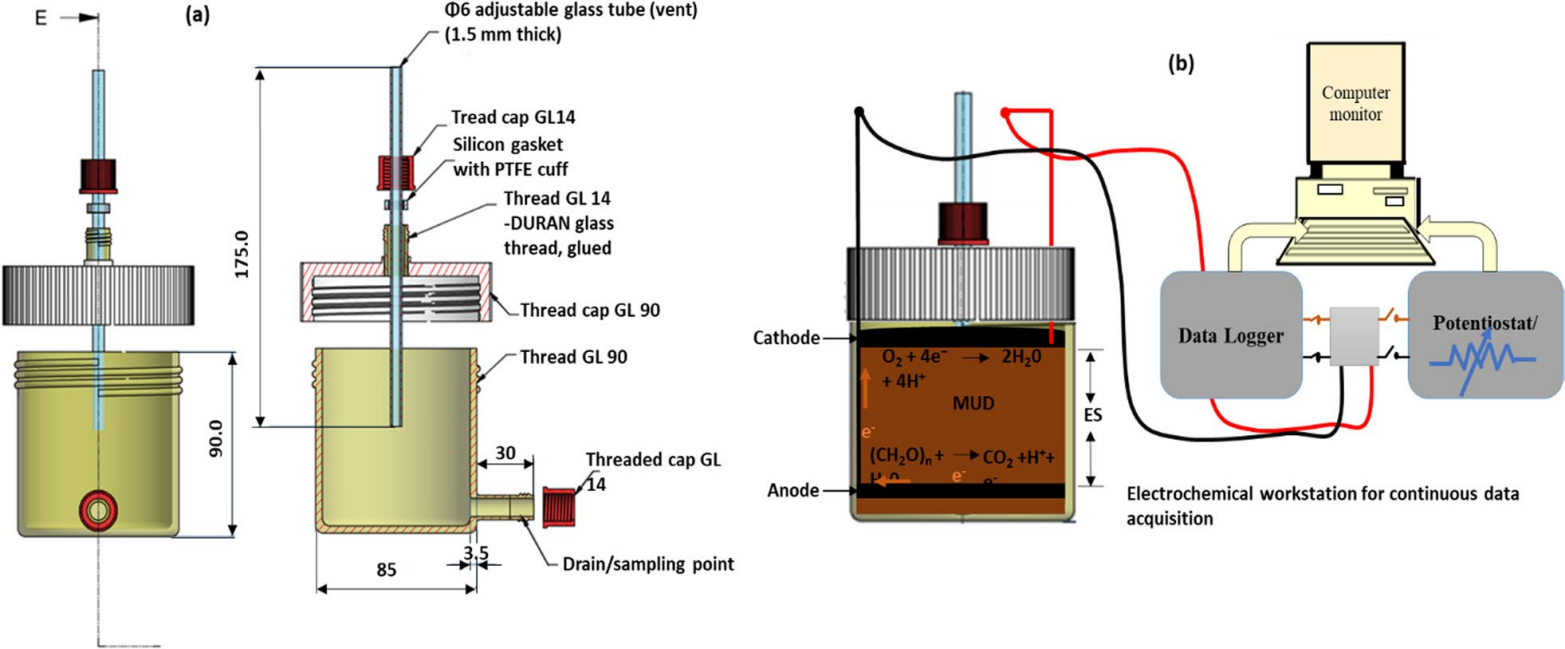
**a** Design of MFC reactors; (**b**) complete Experimental setup for continuous data capturing

CF electrodes (AvCarb C100 soft CF) were purchased from the fuel cell store (https://www.fuelcellstore.com). After heat treatment at 400 °C, each CF electrode was connected to a titanium wire current collector (0.5 mm diameter, Mateck, Germany). SEC electrodes were made by integrating a stainless-steel wire mesh, carbon-black (CB) (Vulcan-72), and a binder (two-component epoxy) into one unit as previously described [48], each electrode was prepared into circular slices of radius 3.25 cm. The thickness (t) of the CF electrode was 3.2 mm, while that of the SEC was 1.7 mm. The MFCs were assembled as previously described [4, 27] and demonstrated in Fig. 8b. The soil slurry used was biologically active soil with a good mixture of sand, silt, and clay that was systematically prepared by saturating with water. The composition of the sand in the mixture was above 50% to allow the substrate to flow easily into the anode region [21]. A layer of soil slurry about 1 cm thick was applied to the bottom of the MFC, on top of which additional layers (2 cm, 4 cm, and 8 cm) were applied according to the ES. Synthetic substrate [4, 49] was fed to the MFCs according to the designed SFI when the natural substrate of the soil was exhausted. Before each feeding, excess moisture or substrate from the previous feeding was either removed through the sampling port at the base of the MFCs or carefully drained from the top. The first substrate addition (feeding) was done when a drop in performance was observed in most MFCs after a period of exponential voltage growth monitored by a data logger (ADC-24). Thus, the first feeding was done after 18 days of operation. Before this point, the MFCs were only occasionally fed tap water to compensate for moisture loss through evaporation.

### Determination of electrochemical and physicochemical properties of the electrodes

Linear sweep voltammetry (LSV) with a scan rate of 1 mV/s was used to extract the electrochemical power indices, by tracking the maximum power point (MPP) from the MFCs and monitoring the performance of the system over time. The LSV was repeated at least every three (3) days from no-load potential to zero potential [4]. This was necessary to monitor how the different experimental treatments affect the maximum performance of the MFCs based on a single estimate, and to observe the long-term effects of the combined experimental treatments on the overall performance and stability of the MFCs. Using Electrochemical Lab's fuel cell analysis tool (EC-lab V11.32), the MPP parameters (current (I), voltage (V), and power (P)) were determined from the power and polarisation curves. The external resistance (${R}_{ex}$) at maximum power was calculated from Ohm's law (R = V/I). The measured cell voltage (V) of the MFCs was considered as a linear function of the current according to the following equation:

2 $$\mathrm{OCV}=V+{\mathrm{Ir}}_{\mathrm{int}}$$

$\mathrm{OCV}$ is the open-circuit voltage and ${\mathrm{Ir}}_{\mathrm{int}}$ is the sum of all the overpotentials of the MFC. By rearranging Eq. 2, the total internal resistance (${R}_{int}$) was computed according to the following equation:

3 $${\mathrm{R}}_{\mathrm{int }}= \frac{\mathrm{OCV}-{\mathrm{E}}_{\mathrm{cell}}}{\mathrm{I}} \mathrm{or }\left(\frac{OCV}{V}-1\right)*{R}_{ex}$$

**Fig. 9:**
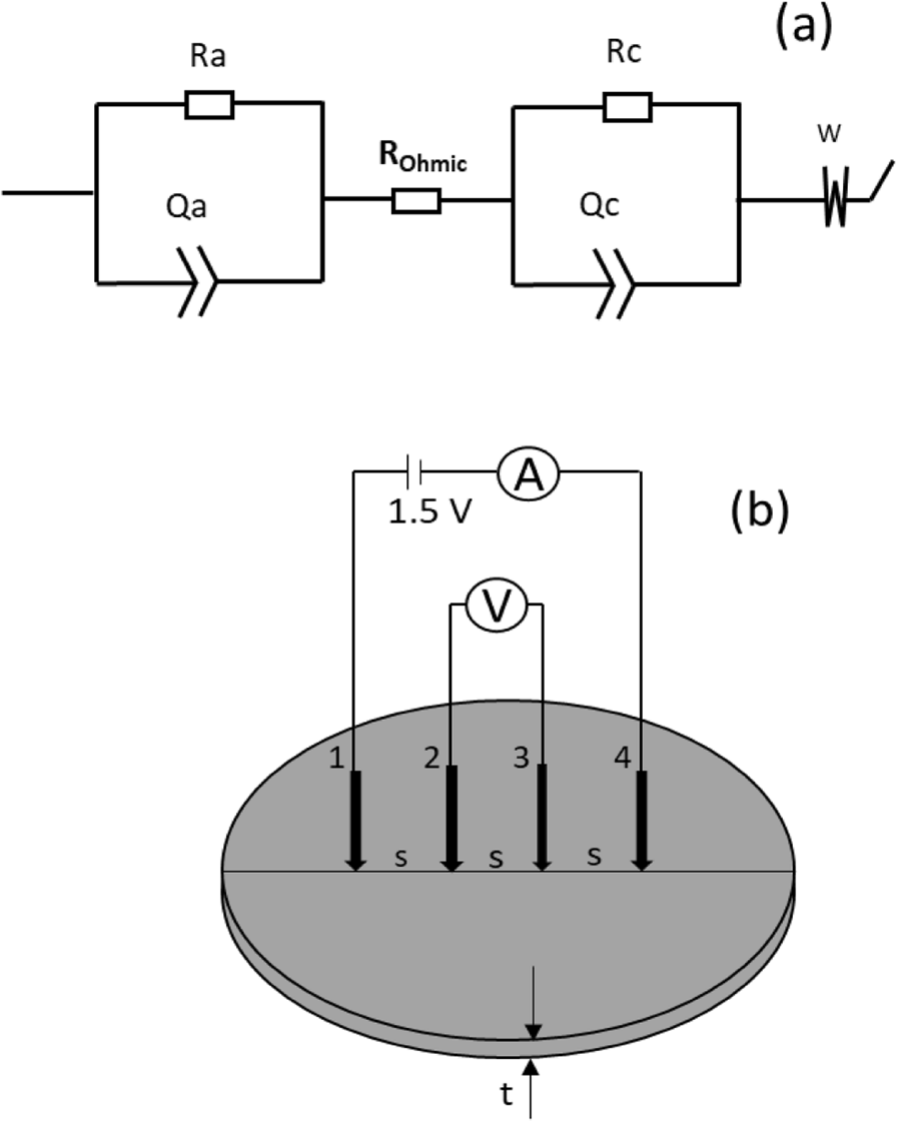
**a** Electrical equivalent circuit for fitting the EIS parameters from Nyquist plot (Additional file 1: Figs. S2, S3 of the supplementary document). **b** Determination of electrode resistivity using the four-point probe method: A is the multimeter for current measurement, V is the multimeter for voltage measurement *s* = 9.4 mm is the distance between the probes, and t is the thickness of the electrode. 1, 2, 3, and 4 represent the probes

Electrochemical impedance spectroscopy (EIS) was performed at an amplitude of 10 mV and a frequency range of 100 kHz to 10 MHz on the best performing MFCs during steady-state operation to study the effects of electrodes on the overall impedance kinetics of the S-MFC. For the modeling of the physical systems with an electrical equivalent circuit, a full cell model with two identical electrodes [50] in a semi-solid electrolyte (mud) was assumed (since the anode and cathode were identical), considering the kinetics of the porous electrodes in the presence of redox species [27]. Only circuit elements that represent physical components of the SMFC configuration used in the study were selected. Therefore, the circuit (C_a_/R_a_ + R_Ohmic_ + C_c_/R_c_ + W) circuit was chosen, so that the diffuse double layer capacitance of the anode (C_a_) and cathode (C_c_) were connected parallel (/) to their respective charge transfer resistance, R_a_ and R_c_. The uncompensated electrolyte (mud) resistance (R_Ohmic_) was connected in series ( +), in addition to the equivalent Warburg impedance (W), which was added to model the diffusion processes taking place in the whole cell at low frequencies. The electrical equivalent circuit is shown schematically in Fig. 9 a. As the electrodes are not ideal capacitance, constant phase elements (CPE or Q) were used in place of the capacitance. The capacitance values were obtained through the “PseudoC” tool of EC-lab or calculated from Eq. 4. Both EIS and LSV were performed using a Potentiostat (Biologic VMP3, France).

4 $$C=\frac{(Q*{R)}^\frac{1}{n}}{R}$$

Q, n, and R are the values resulting from the least square fit of the EIS data to the electrical equivalent circuit; n is the coefficient of the constant phase element. Since a two-electrode system was used to measure the EIS parameters, the equivalent capacitance of the full cell in farads [C (F)] was calculated according to Eq. 5, considering the anode and cathode as two pseudo-capacitors connected in series. Similarly, the total charge transfer resistance [R_ct_ (Ω)] was determined by adding R_a_ and R_c_.

5 $$C\left(F\right)=\frac{{(C}_{a}*{C}_{c})}{({C}_{a}+{C}_{c})}$$

The specific surface area of the electrodes was determined by Brunauer–Emmett–Teller (BET) nitrogen adsorption using the Quantachrome device (Autosorb iQ2) [51]. The resistivity of the electrodes was determined using the four-point probe method (Fig. 9). The probes of two digital multi-meters (DT-830B) were positioned equidistantly (*s* = 9.4 mm) along the diameter of the electrodes to measure the current (I) and voltage (V), as described in Fig. 9. The resistivity (ρ) was calculated according to the following equation:

6 $$\rho =G*\frac{V}{I}$$

where $G$ is the geometric correction factor of the electrodes? In this case, G was calculated from Eq. 7 considering each of the electrodes as an infinitely large thin slice, because t/s <  < 1 for both electrodes [33, 52]:

7 $$G=\frac{\pi }{In2}*t$$

8 $$\rho =\frac{\pi }{In2}*t *\frac{V}{I}$$

### Morphological characteristics of the electrodes and the biofilms

At the end of the experiment, 2 cm^2^ of the anode of the best performing MFC was taken from each EM and prepared according to the protocol modified from Cornejo et al. [53] as follows: the electrode samples were fixed overnight in a 2.5% glutaraldehyde solution in phosphate buffer (pH = 7.0). The samples were then exposed to increasing concentrations of ethanol between 50% and 100% in step increase of 10% for at least 5 min at each concentration. The samples were then dried by immersion in hexamethyldisilazane (HMDS) and absolute ethanol in a 1:1 (v/v) ratio for 10 min, followed by 10 min in 100% HMDS, and finally, the samples were exposed to air for 4 h and placed in a desiccator for 1 h before scanning electron microscopy (SEM) at the Bavarian Polymer Institute, University of Bayreuth, Germany.

### Extraction and purification of nucleic acid

Soil samples were collected directly from the anode and cathode of the MFCs at the MPP (about 40 days) and at the end of the study (90 days). Nucleic acid was extracted from about 0.5 g of the samples using NucleonSpin Soil DNA Purification Kits (Macherey–Nagel, Germany). Extraction and purification from sample preparation to DNA elution followed the protocol provided with the kits, but without the DNA enhancer, or when the enhancer was used, the volume was reduced to 20 µl instead of 150 µl according to the protocol. The quality and concentration of nucleic acid in each sample were determined with NanoDrop 2000 (Thermo Scientific), before sequencing.

### PCR amplification and sequencing of 16S rDNA fragments

16S amplicon libraries of the V4 prokaryotic rDNA genes were constructed from ca. 1–3 ng of metagenomic DNA using primers 515F [54] and 806RB [55] extended with Illumina overhang adapters as described in the 16S Metagenomic Sequencing Library Preparation protocol (Part # 15044223 Rev. B, www.illumina.com). Sample libraries were barcoded using the Nextera XT Index kit (v2 set A, www.illumina.com). All libraries were analyzed by capillary electrophoresis (Fragment Analyzer 5200, www.agilent.com) and combined in equimolar amounts for subsequence sequencing on Illumina’s iSeq-100 platform in custom mode (read 1 set to 293 cycles). Demultiplexing of reads was done on the iSeq-100 platform and the sample-specific reads were saved in FastQ format.

### Bioinformatic analyses

The forward reads of the 16S rDNA fragments were analyzed using the QIIME2 microbiome analysis package (ver. 2021.2, 56). Unless indicated otherwise, all analysis tools were used as plugins within the QIIME2 package. The detailed parameters used for the analysis steps are embedded as provenance information in the QIIME2 data files (available as supplemental data), and the major analysis steps are shortly summarized here: the demultiplexed reads were denoised, dereplicated, and analyzed for chimera sequences using ‘DADA2’ resulting in amplified sequence varians (ASVs). Rare ASVs were filtered out by the median frequency (set to 13) of ASVs over all samples. Taxonomic classification of reads was performed using a pre-fitted sklearn-based taxonomy classifier based on the SILVA reference database (ver. 138, 57, 58) at 99% identity level and limited to simulated amplicons extracted by primer combination 515F/806R (available at docs.qiime2.org). Phylogenetic analyses were calculated using plugin ‘align-to-tree-mafft-fasttree’, which were used to calculate both phylogenetic and non-phylogenetic diversity metrics by plugin ‘core-metric-phylogenetic’. The QIIME2 plugins ‘DEICODE’ and ‘QURRO’ [59, 60] were used to prepare Robust Aitchison PCA (RPCA) plots and to get access to the ranked list of features contributing to the separation of sample along the RPCA ordinations. Significant differences between sample groups were analyzed by plugin ‘beta-group-significance’ using Permanova tests.

## Supplementary Information

**Additional file 1:  Figure S1.** SMFC performance profile at different electrode spacings, substrate feeding intervals and electrode materials. (a) OCV from first block, the arrow shows point of first substrate feeding; (b) OCV from second block; (c) maximum power trends of the two blocks combined. The trends shown with dotted lines represent CF electrodes. Please refer to Table 1 of the main document for details of the different levels of the variables. **Figure S2.** Nyquist plots and the fits of SEC–MFCs at different electrode spacing: 2 h, 4 h and 8 h are SEC–MFCs at electrode spacing (ES) of 2, 4 and 8 cm, respectively. **Figure S3.** Nyquist plots of CF–MFCs at ES of 2, 4 and 8 cm, respectively (N:B Graphs were copied directly from EC-lab V11.32, where the fitting was performed). **Figure S4.** Taxonomic distribution of the 16S rDNA microbial community profile at the Phylum level. **Table S1.** Analysis of variance table for the model design. **Table S2.** Solutions for 2 combinations of categoric factor level. **Table S3.** Pairwise permanova results based on beta-group-significance (α = 0.05).

## Data Availability

Data not provided within the text are available as an Additional file.
