# Supplementary material for: Optimization of soil microbial fuel cell for sustainable bio-electricity production: combined effects of electrode material, electrode spacing, and substrate feeding frequency on power generation and microbial community diversity
Source: Biotechnol Biofuels Bioprod. 2022 Nov 16;15:124. doi: 10.1186/s13068-022-02224-9 (PMC9667596; doi:10.1186/s13068-022-02224-9)
Supplement: Supplementary file 1 — Additional file 1: Figure S1. SMFC performance profile at different electrode spacings, substrate feeding intervals and electrode materials. (a) OCV from first block, the arrow shows point of first substrate feeding; (b) OCV from second block; (c) maximum power trends of the two blocks combined. The trends shown with dotted lines represent CF electrodes. Please refer to Table 1 of the main document for details of the different levels of the variables. Figure S2. Nyquist plots and the fits of SEC–MFCs at different electrode spacing: 2 h, 4 h and 8 h are SEC–MFCs at electrode spacing (ES) of 2, 4 and 8 cm, respectively. Figure S3. Nyquist plots of CF–MFCs at ES of 2, 4 and 8 cm, respectively (N:B Graphs were copied directly from EC-lab V11.32, where the fitting was performed). Figure S4. Taxonomic distribution of the 16S rDNA microbial community profile at the Phylum level. Table S1. Analysis of variance table for the model design. Table S2. Solutions for 2 combinations of categoric factor level. Table S3. Pairwise permanova results based on beta-group-significance (α = 0.05). [file 13068_2022_2224_MOESM1_ESM.docx]

**Additional file**

Optimization of soil microbial fuel cell for sustainable bio-electricity production: Combined effects of electrode material, electrode spacing and substrate feeding frequency on power generation and microbial community diversity

^1,3^Simeon M. Imologie^*^., ^2^Alfons R. Weig, ^1^Freitag R.

FigS1: SMFC performance profile at different electrode spacings, substrate feeding intervals and electrode materials. (a) OCV from first block, the arrow shows point of first substrate feeding; (b) OCV from second block; (c) maximum power trends of the two blocks combined. The trends shown with dotted lines represent CF electrodes. Please refer to Table 1 of the main document for details of the different levels of the variables

FigS2. Nyquist plots and the fits of SEC-MFCs at different electrode spacing: 2h, 4h and 8h are SEC-MFCs at electrode spacing (ES) of 2, 4 and 8 cm respectively

FigS3 Nyquist plots of CF-MFCs at ES of 2, 4 and 8 cm respectively (N:B Graphs were copied directly from EC-lab V11.32 where the fitting was performed)

Sample calculation for the EIS parameters

**SEC-8, for instance (**(**please refer to Table 2 of the main document**):

| === Z fit Analysis (07.21.22 19:17) === | | | |
| --- | --- | --- | --- |
| Equivalent circuit: | | |  |
| R1+Q2/R2+Q3/R3+W3 | | |  |
| R1 = 70,22 Ohm | | |  |
| Q2 = 0,858 9e-3 F.s^(a - 1) | | | |
| a2 or n = 0,475 4 | |  |  |
| R2 = 8,823 Ohm | | |  |
| Q3 = 0,868 2e-3 F.s^(a - 1) | | | |
| a3 or n = 0,710 5 | |  |  |
| R3 = 36,65 Ohm | | |  |
| s3 or σ = 3,152 Ohm.s^-1/2 | | |  |
| Fit: | |  |  |
| select: current cycle(s) | | |  |
| method: Randomize + Simplex | | | |
| stop Randomize on: 100000 iterations | | | |
| stop fit on: 100000 iterations | | | |
| weight: 1  Actual values obtained with the “PseudoC“ of EC-lab or calculated according to **equation 4** | |  |  |
| Equivalent circuit: |  |  |  |
| R1+C2/R2+C3/R3+W3 |  |  |  |
| R1 = 70.22 Ohm |  |  |  |
| C2 = 3.927e-6 F |  |  |  |
| R2 = 8.823 Ohm |  |  |  |
| C3 = 0.213 1e-3 F |  |  |  |
| R3 = 36.65 Ohm |  |  |  |
| s3 = 3.152 Ohm.s^-1/2 |  |  |  |

Considering the electrodes as resistors in series (for a full-cell measurement)

R_ct_ = R2+R3 = 45.473Ω

C(Farad) = (c2*C3)/(c2+c3) = 3.86E-06

**Table S1 Analysis of variance table for the model design**

| Response | 1 | Power |  |
| --- | --- | --- | --- |
| Transform: | Square Root | Constant: | 0 |

| **ANOVA for Response Surface Quadratic model** | | | | | | |
| --- | --- | --- | --- | --- | --- | --- |
| **Analysis of variance table [Partial sum of squares - Type III]** | | | | | | |
|  | **Sum of** |  | **Mean** | **F** | **p-value** |  |
| **Source** | **Squares** | **df** | **Square** | **Value** | **Prob > F** |  |
| Block | 7.051E-003 | 1 | 7.051E-003 |  |  |  |
| Model | 0.85 | 8 | 0.11 | 22.13 | 0.0001 | significant |
| *A-ES* | *1.921E-003* | *1* | *1.921E-003* | *0.40* | *0.5452* |  |
| *B-FF* | *0.019* | *1* | *0.019* | *3.89* | *0.0842* |  |
| *C-Electrode type* | *0.37* | *1* | *0.37* | *76.72* | *< 0.0001* |  |
| *AB* | *2.188E-003* | *1* | *2.188E-003* | *0.45* | *0.5192* |  |
| *AC* | *6.534E-003* | *1* | *6.534E-003* | *1.36* | *0.2776* |  |
| *BC* | *6.295E-003* | *1* | *6.295E-003* | *1.31* | *0.2859* |  |
| *A^2^* | *0.042* | *1* | *0.042* | *8.72* | *0.0183* |  |
| *B^2^* | *0.013* | *1* | *0.013* | *2.80* | *0.1328* |  |
| Residual | 0.039 | 8 | 4.815E-003 |  |  |  |
| Cor Total | 0.90 | 17 |  |  |  |  |

The Model F-value of 22.13 implies the model is significant. There is only

a 0.01% chance that an F-value this large could occur due to noise.

Values of "Prob > F" less than 0.0500 indicate model terms are significant.

In this case C, A^2 are significant model terms.

Values greater than 0.1000 indicate the model terms are not significant.

If there are many insignificant model terms (not counting those required to support hierarchy),

model reduction may improve your model.

| Std. Dev. | 0.069 |  | R-Squared | 0.9568 |
| --- | --- | --- | --- | --- |
| Mean | 0.39 |  | Adj R-Squared | 0.9135 |
| C.V. % | 17.62 |  | Pred R-Squared | 0.7492 |
| PRESS | 0.22 |  | Adeq Precision | 12.764 |
| -2 Log Likelihood | -59.56 |  | BIC | -30.66 |
|  |  |  | AICc | -8.14 |

The "Pred R-Squared" of 0.7492 is in reasonable agreement with the "Adj R-Squared" of 0.9135;

i.e. the difference is less than 0.2.

"Adeq Precision" measures the signal to noise ratio. A ratio greater than 4 is desirable. Your

ratio of 12.764 indicates an adequate signal. This model can be used to navigate the design space.

|  | **Coefficient** |  | **Standard** | **95% CI** | **95% CI** |  |
| --- | --- | --- | --- | --- | --- | --- |
| **Factor** | **Estimate** | **df** | **Error** | **Low** | **High** | **VIF** |
| Intercept | 0.43 | 1 | 0.040 | 0.34 | 0.53 |  |
| Block 1 | 2.140E-003 | 1 |  |  |  |  |
| Block 2 | -2.140E-003 |  |  |  |  |  |
| A-ES | 0.020 | 1 | 0.031 | -0.052 | 0.092 | 2.54 |
| B-SFI | 0.14 | 1 | 0.072 | -0.024 | 0.31 | 13.02 |
| C-Electrode type | 0.23 | 1 | 0.026 | 0.17 | 0.29 | 2.53 |
| AB | 0.016 | 1 | 0.024 | -0.039 | 0.072 | 2.52 |
| AC | -0.023 | 1 | 0.020 | -0.068 | 0.022 | 1.02 |
| BC | -0.023 | 1 | 0.020 | -0.069 | 0.023 | 2.50 |
| A^2^ | -0.12 | 1 | 0.040 | -0.21 | -0.026 | 1.04 |
| B^2^ | -0.058 | 1 | 0.035 | -0.14 | 0.022 | 13.00 |

| **Final Equation in Terms of Coded Factors:** | |
| --- | --- |
| Sqrt(Power) | = |
| +0.43 |  |
| +0.020 | * A |
| +0.14 | * B |
| +0.23 | * C |
| +0.016 | * AB |
| -0.023 | * AC |
| -0.023 | * BC |
| -0.12 | * A^2^ |
| -0.058 | * B^2^ |

The equation in terms of coded factors can be used to make predictions about the response for

given levels of each factor. By default, the high levels of the factors are coded as +1 and the

low levels of the factors are coded as -1. The coded equation is useful for identifying the

relative impact of the factors by comparing the factor coefficients.

| **Final Equation in Terms of Actual Factors:** | |
| --- | --- |
| Electrode type | carbon felt |
| Sqrt(Power) | = |
| -0.69944 |  |
| +0.13383 | * ES |
| +0.18525 | * SFI |
| +2.70682E-003 | * ES * SFI |
| -0.013041 | * ES^2^ |
| -0.014512 | * SFI^2^ |
| Electrode type | stainless |
| Sqrt(Power) | = |
| -0.075406 |  |
| +0.11853 | * ES |
| +0.16235 | * SFI |
| +2.70682E-003 | * ES * SFI |
| -0.013041 | * ES^2^ |
| -0.014512 | * SFI^2^ |

The equation in terms of actual factors can be used to make predictions about the response for

given levels of each factor. Here, the levels should be specified in the original units for

each factor. This equation should not be used to determine the relative impact of each factor

because the coefficients are scaled to accommodate the units of each factor and the intercept

is not at the center of the design space.

| **Table S2: Solutions for 2 combinations of categoric factor levels** | | | | | | | |
| --- | --- | --- | --- | --- | --- | --- | --- |
| **Number** | **ES (cm)** | **SFI (day)** | **Electrode type** | **Power (µW)** | **StdErr(Sqrt(Power))** | **Desirability** |  |
| 1 | 4.309 | 7.389 | SEC | 0.476 | 0.040 | 0.938 | Selected |
| 2 | 6.365 | 7.185 | SEC | 0.483 | 0.042 | 0.905 |  |
| 3 | 6.354 | 7.231 | CF | 0.113 | 0.042 | 0.548 |  |


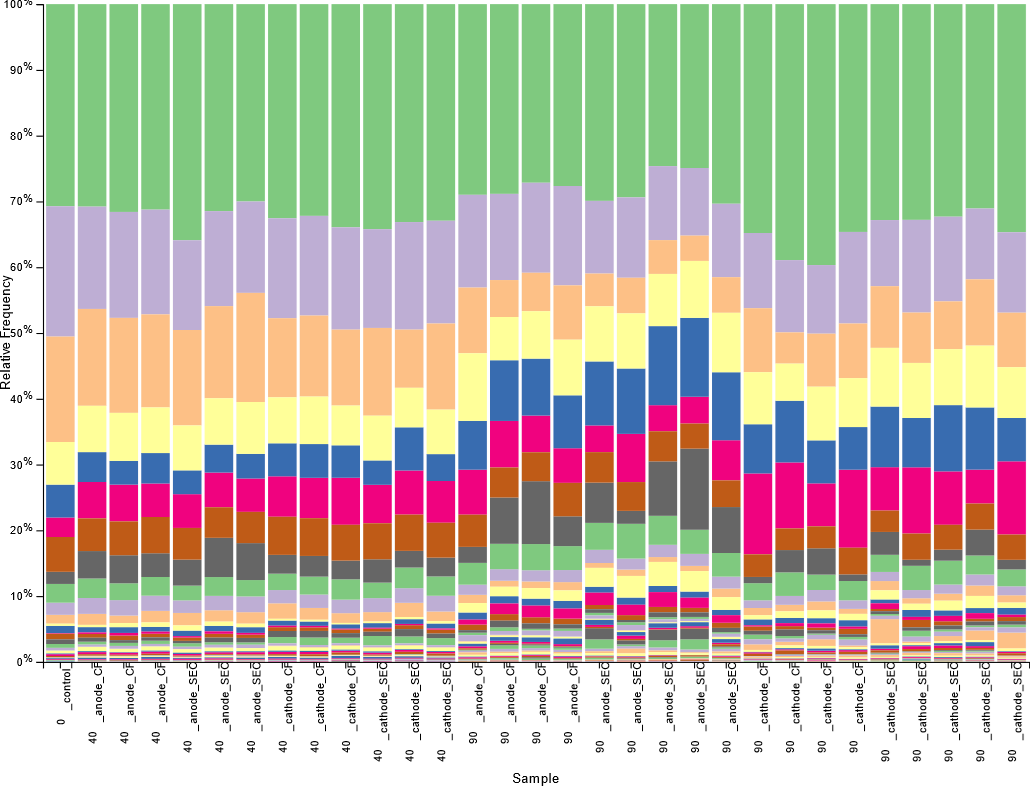

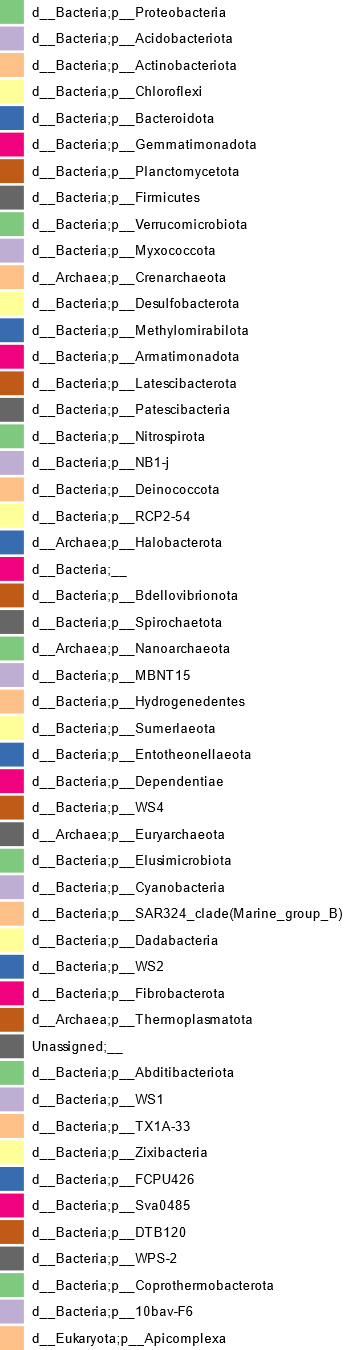


FigS4: Taxonomic distribution of the 16S rDNA microbial community profile at the Phylum level

Table S3: Pairwise permanova results based on beta-group-significance (α = 0.05)

|  | Group 2 | Sample size | Permutations | pseudo-F | p-value | q-value |  |
| --- | --- | --- | --- | --- | --- | --- | --- |
| 40_anode_CF | 40_anode_SEC | 6 | 999 | 1.111 | 0.496 | 0.51 | 0.51 |
|  | 40_cathode_CF | 6 | 999 | 18.814 | 0.088 | 0.147 | 0.147 |
|  | 40_cathode_SEC | 6 | 999 | 7.729 | 0.09 | 0.147 | 0.147 |
|  | 90_anode_CF | 7 | 999 | 22.293 | 0.025 | 0.06 | 0.06 |
|  | 90_anode_SEC | 8 | 999 | 24.141 | 0.019 | 0.06 | 0.06 |
|  | 90_cathode_CF | 7 | 999 | 40.341 | 0.032 | 0.064 | 0.064 |
|  | 90_cathode_SEC | 8 | 999 | 72.545 | 0.019 | 0.06 | 0.06 |
| 40_anode_SEC | 40_cathode_CF | 6 | 999 | 7.688 | 0.095 | 0.149 | 0.149 |
|  | 40_cathode_SEC | 6 | 999 | 3.562 | 0.19 | 0.255 | 0.255 |
|  | 90_anode_CF | 7 | 999 | 19.953 | 0.023 | 0.06 | 0.06 |
|  | 90_anode_SEC | 8 | 999 | 23.025 | 0.019 | 0.06 | 0.06 |
|  | 90_cathode_CF | 7 | 999 | 31.722 | 0.037 | 0.067 | 0.067 |
|  | 90_cathode_SEC | 8 | 999 | 56.756 | 0.018 | 0.06 | 0.06 |
| 40_cathode_CF | 40_cathode_SEC | 6 | 999 | 2.149 | 0.193 | 0.255 | 0.255 |
|  | 90_anode_CF | 7 | 999 | 33.881 | 0.035 | 0.066 | 0.066 |
|  | 90_anode_SEC | 8 | 999 | 34.866 | 0.02 | 0.06 | 0.06 |
|  | 90_cathode_CF | 7 | 999 | 38.827 | 0.029 | 0.061 | 0.061 |
|  | 90_cathode_SEC | 8 | 999 | 72.907 | 0.018 | 0.06 | 0.06 |
| 40_cathode_SEC | 90_anode_CF | 7 | 999 | 30.162 | 0.028 | 0.061 | 0.061 |
|  | 90_anode_SEC | 8 | 999 | 31.503 | 0.022 | 0.06 | 0.06 |
|  | 90_cathode_CF | 7 | 999 | 39.39 | 0.024 | 0.06 | 0.06 |
|  | 90_cathode_SEC | 8 | 999 | 72.854 | 0.02 | 0.06 | 0.06 |
| 90_anode_CF | 90_anode_SEC | 9 | 999 | 1.38 | 0.29 | 0.316 | 0.316 |
|  | 90_cathode_CF | 8 | 999 | 21.442 | 0.025 | 0.06 | 0.06 |
|  | 90_cathode_SEC | 9 | 999 | 28.741 | 0.008 | 0.06 | 0.06 |
| 90_anode_SEC | 90_cathode_CF | 9 | 999 | 28.497 | 0.011 | 0.06 | 0.06 |
|  | 90_cathode_SEC | 10 | 999 | 36.308 | 0.01 | 0.06 | 0.06 |
| 90_cathode_CF | 90_cathode_SEC | 9 | 999 | 0.353 | 0.553 | 0.553 | 0.553 |
